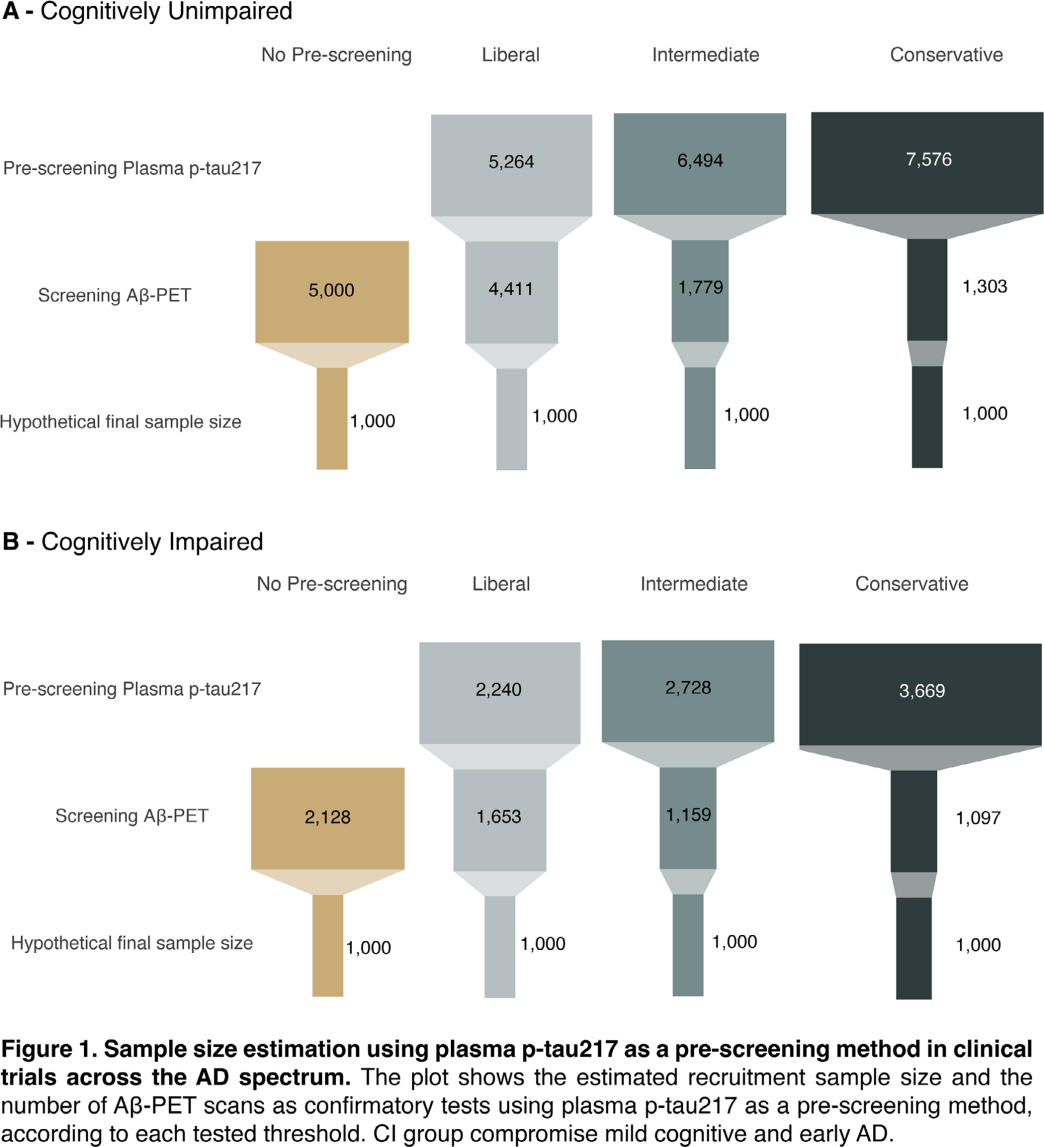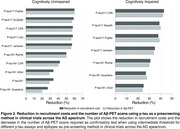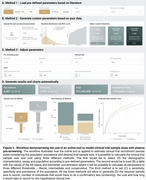# Reference charts of plasma *p*‐tau217 as a pre‐screening tool in AD clinical trials

**DOI:** 10.1002/alz70862_110204

**Published:** 2025-12-23

**Authors:** Pamela C.L. Ferreira, Guilherme Povala, Bruna Bellaver, Guilherme Bauer‐Negrini, Cristiano Aguzzoli, Firoza Z Lussier, João Pedro Ferrari‐Souza, Douglas Teixeira Leffa, Marina Scop Madeiros, Carolina Soares, Helmet T. Karim, Eduardo R. Zimmer, Chang Hyung Hong, Hyun Woong Roh, Ann D Cohen, Pedro Rosa‐Neto, Dana L Tudorascu, Beth E. Snitz, Thomas K Karikari, Sterling C Johnson, Sang Joon Son, Tharick A Pascoal

**Affiliations:** ^1^ University of Pittsburgh, Pittsburgh, PA USA; ^2^ Brain Institute of Rio Grande do Sul (InsCer), Porto Alegre, Rio Grande do Sul Brazil; ^3^ Universidade Federal do Rio Grande do Sul, Porto Alegre, RS Brazil; ^4^ UFRGS, Porto Alegre Brazil; ^5^ Ajou University School of Medicine, Suwon, Gyeonggido Korea, Republic of (South); ^6^ McGill University, Montreal, QC Canada; ^7^ University of Pittsburgh School of Medicine, Pittsburgh, PA USA; ^8^ University of Wisconsin‐Madison, Madison, WI USA

## Abstract

**Background:**

Plasma phosphorylated tau (*p*‐tau) has been used as a pre‐screening tool in clinical trials. Using plasma *p*‐tau for pre‐screening may help identify individuals more likely to be Aβ‐PET positive, reducing the number of PET scans needed at enrollment and decreasing recruitment costs. This study aims to evaluate the cost‐effectiveness of plasma *p*‐tau using liberal, intermediate, and conservative thresholds as pre‐screening tools in clinical trials across the AD spectrum.

**Method:**

We studied 1,673 individuals across the AD spectrum from five cohorts: 707 cognitively unimpaired(CU) and 966 cognitively impaired(CI) with plasma *p*‐tau217 measures and Aβ‐PET. We tested three threshold methods:liberal(95% sensitivity), intermediate(Youden index), and conservative(95% specificity). The recruitment sample size was calculated based on *p*‐tau217 assay sensitivity, specificity, and Aβ positivity prevalence for a final sample size of 1,000 based on recent phase 3 trials.

**Result:**

The intermediate threshold was the most cost‐effective, followed by the conservative. Surprisingly, the liberal threshold did not outperform the strategy of screening only with Aβ‐PET (Figure 1). Sample Size: In CU, pre‐screening with the intermediate threshold increased the initial sample size by 30% compared to the non‐pre‐screened group (*n* = 5,000), while in CI, it increased by 28%(*n* = 2,128). The number of Aβ‐PET scans was reduced by 64% in CU and 46% in CI. Trial Cost: In CU, the intermediate threshold reduced costs by 59% compared to no pre‐screening($24M). In CI, costs were reduced by 39% compared to no pre‐screening($9.4M). We tested the pre‐screening method using other *p*‐tau217 assays and epitopes and observed reductions in Aβ‐PET scans and recruitment costs for all *p*‐tau assays (Figure 2). Using our models and data, we developed a free online tool that allows users to calculate the sample size needed when using plasma *p*‐tau as a pre‐screening method for recruiting individuals for clinical trials. This tool will be released with our publication at AAIC (Figure 3).

**Conclusion:**

Our study demonstrates that plasma *p*‐tau217 is an effective pre‐screening tool for clinical trials, with the intermediate threshold being the most cost‐effective for both CU and CI. This approach reduces the need for Aβ‐PET scans and optimizes recruitment time and costs, potentially enhancing the efficiency of clinical trial designs within the AD spectrum.